# *sym*-Trisubstituted 1,3,5-Triazine Derivatives as Promising Organic Corrosion Inhibitors for Steel in Acidic Solution

**DOI:** 10.3390/molecules21040436

**Published:** 2016-03-31

**Authors:** Ayman El-Faham, Kholood A. Dahlous, Zeid A. AL Othman, Hamad A. Al-Lohedan, Gamal A. El-Mahdy

**Affiliations:** 1 Department of Chemistry, College of Science, King Saud University, P. O. Box 2455, Riyadh 11451, Saudi Arabia; kdahloos@ksu.edu.sa (K.A.D.); zaothman@ksu.edu.sa (Z.A.A.O.); hlohedan@ksu.edu.sa (H.A.A.-L.); 2 Chemistry Department, Faculty of Science, Alexandria University, P. O. Box 426, Ibrahimia, 12321 Alexandria, Egypt; 3 Advanced Materials Research Chair, Department of Chemistry, College of Science, King Saud University, P. O. Box 2455, Riyadh 11451, Saudi Arabia; 4 Surfactants Research Chair, Department of Chemistry, College of Science, King Saud University, Riyadh 11451, Saudi Arabia; 5 Chemistry Department, Faculty of Science, Helwan University, Helwan 11795, Egypt; Gamalmah2000@yahoo.com

**Keywords:** trisubstituted-*s*-triazine, organic inhibitors, corrosion, steel, polarization

## Abstract

Triazine derivatives, namely, 2,4,6-tris(quinolin-8-yloxy)-1,3,5-triazine (**T3Q**), *N*^2^*,N*^4^*,N*^6^-tris(pyridin-2-ylmethyl)-1,3,5-triazine-2,4,6-triamine (**T3AMPy**) and 2,2′,2′′-[(1,3,5-triazine-2,4,6-triyl)tris(azanediyl)] tris(ethan-1-ol) (**T3EA**) were synthesized and their inhibition of steel corrosion in hydrochloric acid solution was investigated using electrochemical techniques. The corrosion protection of the prepared compounds increased with increasing concentration and reached up to 98% at 250 ppm. The adsorption of **T3Q**, **T3AMPy**, and **T3EA** on the steel surface was in accordance with the Langmuir adsorption isotherm. The electrochemical results revealed that **T3Q**, **T3AMPy** and **T3EA** act as excellent organic inhibitors and can labeled as mixed type inhibitors. The efficiencies of the tested compounds were affected by the nature of the side chain present in the triazine ring, where **T3EA** gave the least inhibition while **T3Q** and **T3AMPy** gave higher and almost the same inhibition effects. The inhibition efficiencies obtained from the different electrochemical techniques were in good agreement.

## 1. Introduction

The use of inhibitors to protect steel from corrosion, mainly in acidic solutions, is one of the most practical methods [1,2,3]. Acids such as HCl, [4,5,6] HNO_3_, and H_2_SO_4_ [7] are usually used for the elimination of metal scale and rust in many manufacturing processes. Because of their aggressive effect on the metal surface, inhibitors are comprehensively used to decrease the corrosion of the metal [8,9]. Organic compounds, especially those composed of nitrogen, oxygen and sulfur atoms, represent the majority of inhibitors used in industry [10,11,12,13]. Inhibitors which contain multiple (double or triple) bonds play an important role in the adsorption and the interaction of these compounds onto the metal surfaces [2]. The adsorption of the organic molecules [14], the charge density [15,16], the mode of adsorption [17,18], the exposal area [19] of the organic inhibitors and the molecular weight could all affect the efficiency of an inhibitor [20]. Organic compounds generally inhibit the corrosion of the metal by forming a film on the metal surface. The degree of inhibition depends on the chemical composition, the structure and the ability of the inhibitor to adsorb on the metal surface. The adsorption of these compounds is influenced by the their electronic structure, steric factors, aromatic character and electron density of the functional groups such as –NH, –N=N–, –C=N, –CHO, R–OH, *etc.* located at the donor site such [20,21,22,23,24,25].

**Figure 1:**
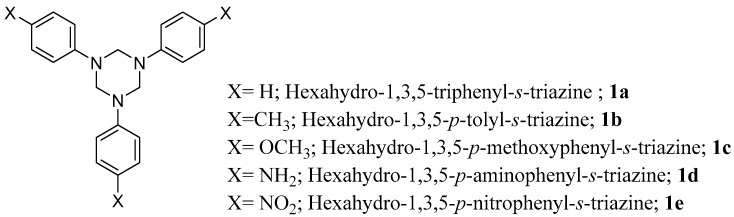
Structures and names of compounds **1a**–**e** as corrosion inhibitors [26].

Recently Shukla *et al.* [26] reported the hexahydro-*s*-triazine derivatives **1a**–**e** (Figure 1) as corrosion inhibitors of steel in 1 N HCl and the inhibition effect depend on the electronic nature of the functional groups present in the compounds.

**Scheme 1:**
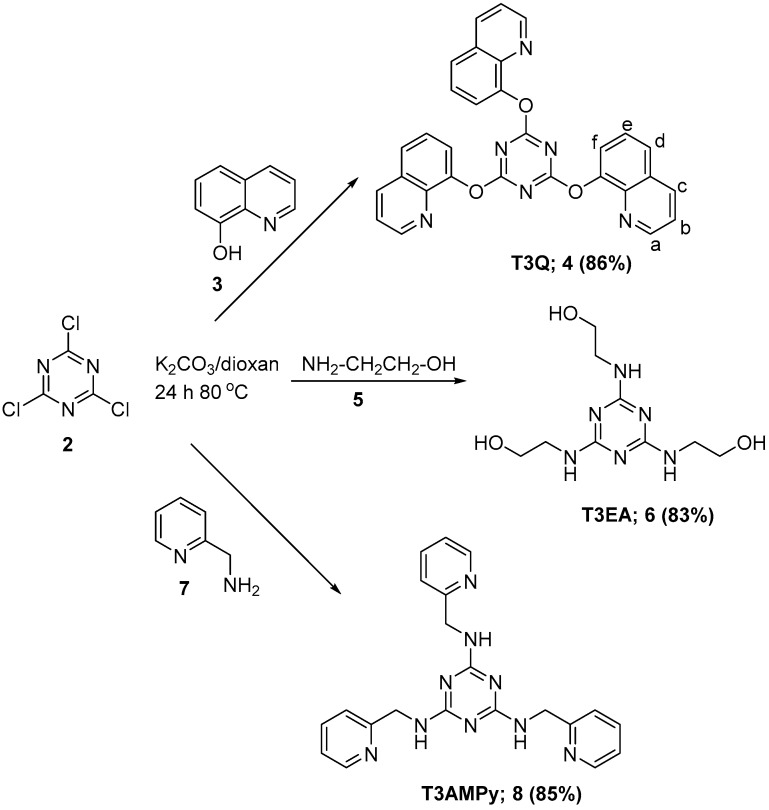
Synthesis of the organic inhibitors **4**, **6**, and **8**.

The work we report here describes a one step and an easy method for the synthesis of three *sym*-trisubstituted 1,3,5-triazine derivatives T3Q, T3AMP**y** and T3EA with different terminal side chains on the triazine ring (Scheme 1) and their application as promising novel organic corrosion inhibitors for steel in HCl solutions.

## 2. Results and Discussion

### 2.1. Synthesis of sym-Trisubstituted-s-triazines ***4***, ***6***, and ***8***

The three inhibitors T3Q (**4**), T3EA (**6**), and T3AMPy (**8**) were prepared by the reaction of nucleophiles **3**, **5**, or **7** with cyanuric chloride (**2**) at 0 °C for 2 h and then the temperature of the reaction mixtures was increased to 80 °C and kept at this temperature for 24 h to afford the target products in 83%–86% yield as shown in Scheme 1. All the spectral data were in good agreement with their proposed structures and with the reported data for compounds **6** and **8** (Experimental Section).

As an example the obtained ^1^H-NMR spectrum (Figure S1) of the new compound 2,4,6-tris(quinolin-8-yloxy)-1,3,5-triazine (T3Q, **4**), showed doublet of doublets at δ 8.81, 8.34, and 7.81 ppm, corresponding to the three protons Ha, Hc, and Hd, respectively (Scheme 1). The other three protons Hb, He, and Hf appear at δ 7.46–7.55 as a multiplet peak. The ^13^C-NMR of compound **4** showed nine carbon signals in the aromatic region at δ 120.9, 122.1, 126.3, 126.2, 128.9, 136.0, 139.9, 147.0, 150.5, corresponding to the quinolone moiety and a signal at δ 173.6 related to the triazine moiety, respectively.

### 2.2. Potentiodynamic Polarization Measurements

**Figure 2:**
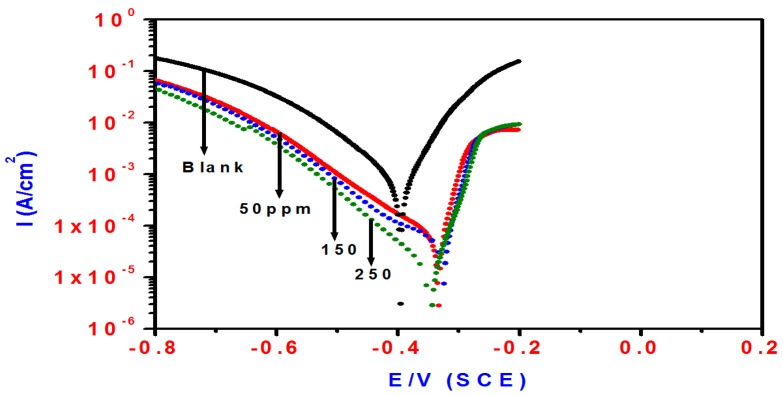
The effect of T3Q concentrations on the polarization curves of steel in acidic chloride solutions.

**Figure 3:**
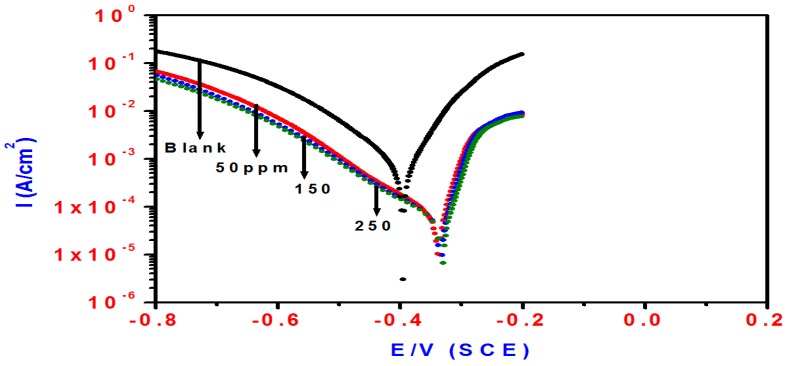
The effect of T3AMPy concentrations on the polarization curves of steel in acidic chloride solutions.

**Figure 4:**
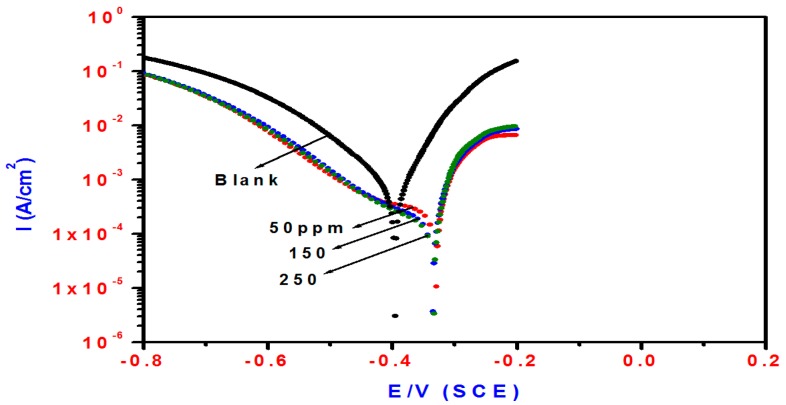
The effect of T3EA concentrations on the polarization curves of steel in acidic chloride solutions.

The effect of the concentration of **4**, **6** and **8** on the polarization curves of steel in acidic chloride solutions is shown in Figure 2, Figure 3 and Figure 4, respectively. These figures show that the addition of **4**, **6**, or **8** to the acidic chloride solution is accompanied by a lowering of the current densities in both directions of the polarization curves. Accordingly, the compounds **4**, **6** and **8** can be labeled as mixed type inhibitors that decrease the anodic and cathodic reactions [27]. All the estimated electrochemical parameters for the compounds **4**, **6**, and **8** are quoted in Table 1. The data clearly shows a dependence of the current density *(Icorr)* on the concentrations of **4**, **6** and **8**. It is obvious that *Icorr* values decreased with increasing inhibitor concentration and the corrosion protection occurred via blocking adsorption of T3Q, T3AMPy and T3EA on the active sites of steel, which enhances the corrosion protection performance of T3Q, T3AMPy and T3EA. The results can be explained on the basis of adsorption of T3Q, T3AMPy and T3EA molecules on the cathodic active sites, which retards the reduction of the H^+^ ion and the corresponding hydrogen evolution by increasing the energy barrier for proton discharge [28,29]. In addition, the anodic dissolution of steel was decreased by the addition of the adsorbed molecules (T3Q, T3AMPy and T3EA) on the anodic active sites. The adsorption of T3Q, T3AMPy and T3EA molecules on the steel surface led to formation of a protective layer and causes a shift in *Ecorr* to more noble [2,30]. Then, we can conclude that the additions of T3Q, T3AMPy and T3EA to the acidic chloride solution caused an enhancement in the corrosion protection performance of steel.

The percentage inhibition efficiency (IE, *%*) can be calculated from:

(1) $$IE\%=\frac{Icorr\left(b\right)-Icorr\left(i\right)}{Icorr\left(b\right)}\times 100$$

where *Icorr (i)* is the corrosion current density in the inhibited and *Icorr (b)* is the corrosion current density in the uninhibited solution. The *IE* values were calculated from Equation (4) and presented in Table 1 for T3Q and T3AMPy. As observed from Table 1, the *IE* is highly dependent on the concentration of the tested materials and increased with increasing concentration of T3Q, T3AMPy and T3EA. The maximum inhibition efficiency (98% for T3Q, 96% for T3AMPy and 85% for T3EA) was attained at a concentration of 250 ppm.

### 2.3. EIS Studies

**Figure 5:**
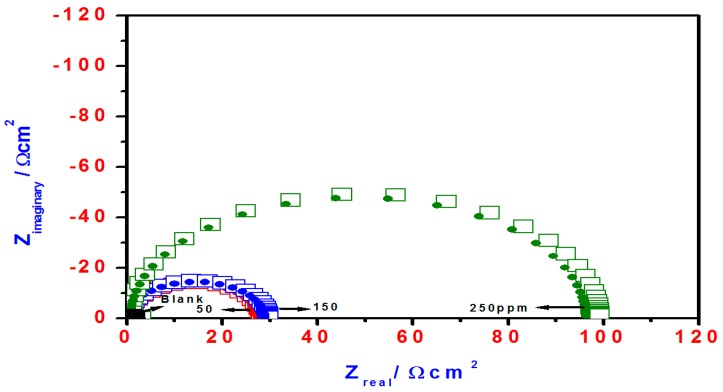
The influence of T3Q concentrations on the the Nyquist plots of steel in acidic chloride solution.

**Figure 6:**
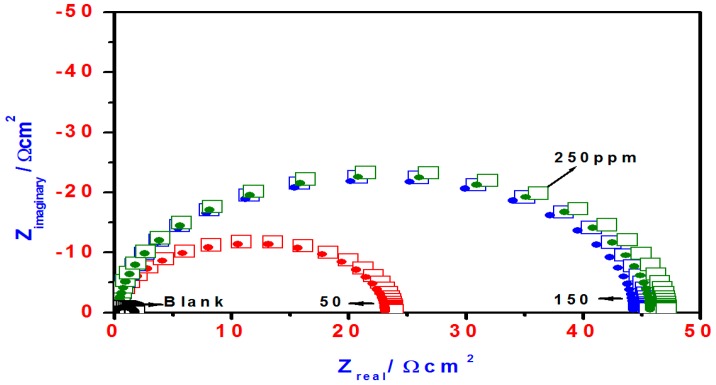
The influence of T3AMPy concentrations on the the Nyquist plots of steel in acidic chloride solution.

**Figure 7:**
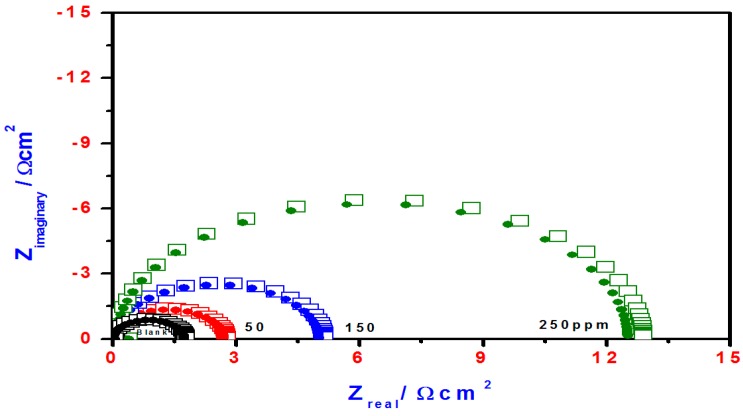
The influence of T3EA concentrations on the the Nyquist plots of steel in acidic chloride solution.

**Figure 8:**
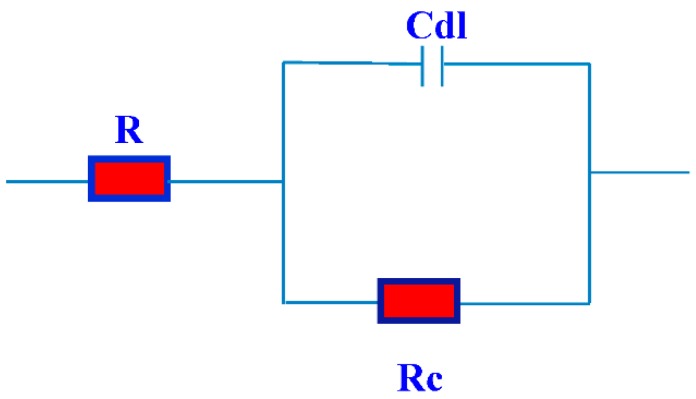
The equivalent used in fitting the EIS experiemtal data.

The influence of T3Q, T3AMPy and T3EA concentrations on the Nyquist plots of steel in acidic chloride solution is shown in Figure 5, Figure 6 and Figure 7, respectively. A capacitive loop is clearly shown in Figure 5, Figure 6 and Figure 7, where the larger the size of the capacitive loops the higher the concentration of T3Q, T3AMPy and T3EA. *EIS* data were fitted by an equivalent circuit (*EC*) comprised of solution resistance (*Rs*), charge transfer resistance (*Rct*) in parallel with double layer capacitance (*Cdl*) as shown in Figure 8. All the impedance parameters for T3Q, T3AMPy and T3EA were estimated and are quoted in Table 1. The percentage inhibition efficiency (*IE*, *%*) as follows:

(2) $$IE\%=\frac{Rct\left(i\right)-Rct\left(b\right)}{Rct\left(i\right)}\times 100$$

where *Rct(i)* and *Rct(b)* are the charge transfer resistances in the inhibited and uninhibited solution, respectively. It is clear that the *IE%* values are strongly dependent upon T3Q, T3AMPy and T3EA concentration and increased with increasing concentration of the tested materials. It seems that the surface of coverage of steel increased with increasing concentration due to an increase in the amount of adsorbed of T3Q, T3AMPY and T3EA on the steel surface, which led to the increase in *IE*. The results are comparable to those obtained from previous studies [31,32]. On the other hand, the value of *Cdl* decreases with the increasing in T3Q, T3AMPy and T3EA concentration. The adsorption of T3Q, T3AMPy and T3EA can be explained by the gradual replacement of water molecules with high dielectric constant by T3Q, T3AMPy and T3EA molecules with low dielectric constant. As immersion time and concentration increased, the thickness of the adsorbed layer increased leading to a decrease in the electrical capacitance [33]. The results of increasing of *Rct* and the decreasing of *Cdl* values suggests that T3Q, T3AMPy and T3EA blocking the active sites on the steel surface via adsorption [34], which could be confirmed that a protective layer formed at the steel/solution, which enhances the corrosion protection performance of steel [35].

### 2.4. Adsorption Isotherm

**Figure 9:**
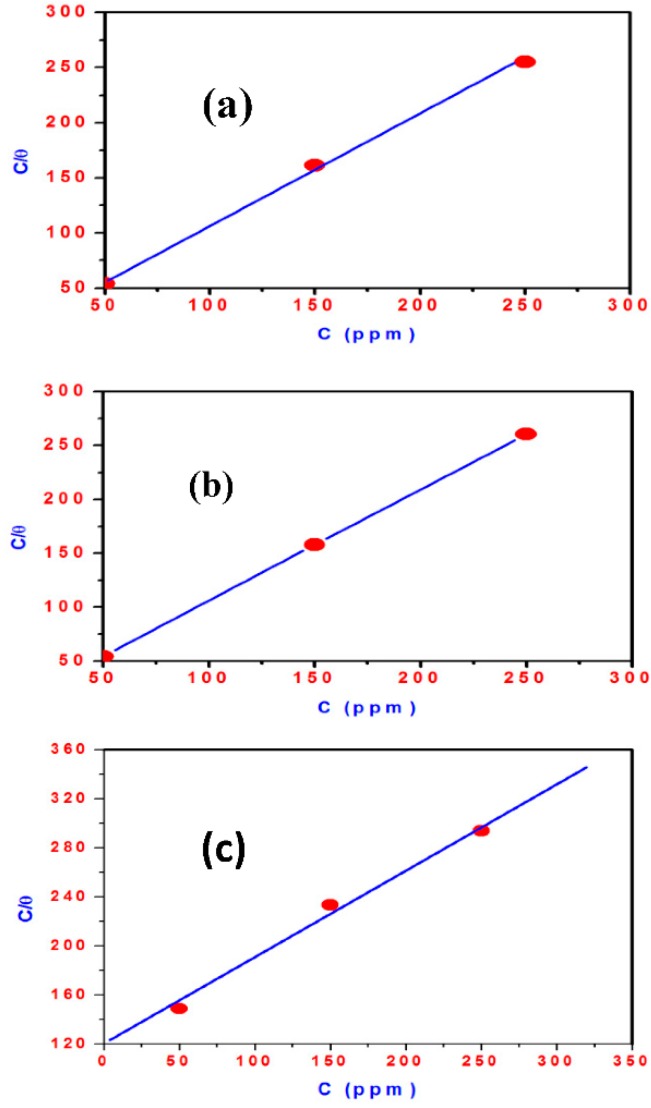
Langmuir adsorption plot of steel in acidic chloride solution containing different concentrations of (**a**) T3Q (**b**) T3AMPy (**c**) T3EA.

The replacement of the pre-adsorbed water molecules by adsorption of the tested materials can be attributed to the interaction energy between the tested materials and the exposed surface. Investigating the different types of adsorption isotherms provide an explanation about the interaction between the steel surface and the T3Q, T3AMPy and T3EA. The experimental data was found to fit well with the Langmuir adsorption isotherm among the various adsorption isotherms. The Langmuir adsorption isotherm related the surface coverage (θ) and the concentration of inhibitor (*Cinh*) as follows [36]:

(3) *C_(inh)_/θ = 1/K_ads_ + C_(inh)_*

where *C_(inh)_* is inhibitor concentration and *Kads* is the equilibrium constant for the adsorption process. The linear relation obtained by plotting *C_(inh)_/θ* *versus* *C_(inh)_* is shown in Figure 9a–c for T3Q, T3AMPy and T3EA, respectively. The regression coefficients were also calculated and found to be 0.999 for T3Q and T3AMPy and 0.99 for T3EA. The calculated value of the slope and the R2 suggests that the adsorption of T3Q, T3AMPy and T3EA on the steel surface follows the Langmuir adsorption isotherm. The standard free energy of adsorption (*ΔG°ads*) and the adsorption constant (*Kads*) are related by the following equation:

(4) *ΔG°ads= −RT ln(55.5Kads)*

**Figure 10:**
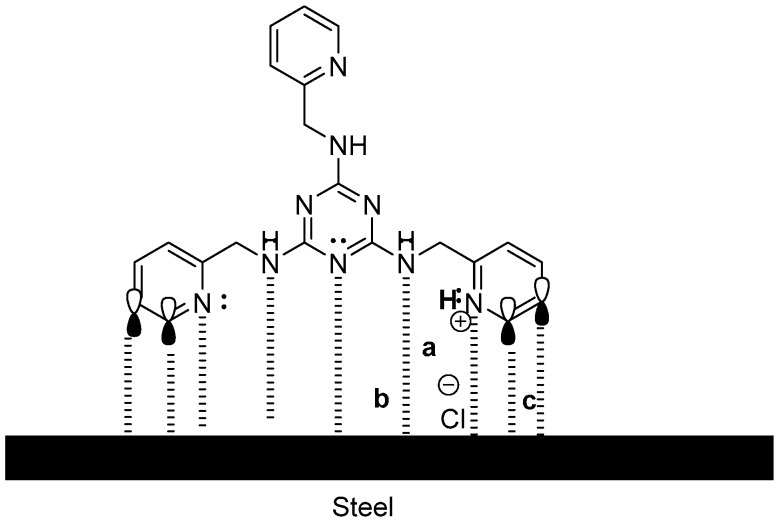
Proposed Schematic representation of the adsorption of the hydrazino-1,3,5-triazines on steel in 1.0 M HCl solution: (**a**) physical adsorption; (**b**) and (**c**) chemical adsorption.

There are two types of adsorption, the first is the physical adsorption and the second one is the chemical adsorption. The former one is predominating when the values of *ΔGads* around −20 kJ·mol^−1^ or lower due to the electrostatic interactions between charged T3Q and TA3MPy and charged steel. The latter process is predominant when the value of *ΔG°ads* around −40 kJ·mol^−1^ or higher through formation of coordinate bond. The estimated values of *ΔG°ads* are −35.65 and −35.85 kJ·mol^−1^ for T3Q and T3AMPy, respectively. In case of T3EA it was found to be −31.87 kJ·mol^−1^. The results suggest that the adsorption mechanism of T3Q, T3AMPy and T3EA on steel surface occurs via physical and chemical adsorption (comprehensive adsorption) [37]. The physical adsorption of the triazine derivatives occurred via electrostatic interactions between the protonated tested materials and the negatively charged steel surface resulted from the adsorption of Cl−anions as shown in Figure 10a. Nitrogen atoms of the triazine ring and hydrazine have unshared electron pairs, which are shared with the empty d-orbital of iron atoms on the steel surface and enhanced the chemical adsorption as shown in Figure 10b. In addition, electron donor−acceptor interactions may also arise between the π-electrons of the imine (C=N) groups of the 1,3,5 triazine ring and the empty d-orbital of iron atoms as shown in Figure 10c. The spontaneity and strong adsoption of T3Q and T3AMPy on steel surface can be accounted the high negative values of *ΔG°ads* [35,38].

## 3. Experimental Section

### 3.1. General Information

The solvents used were of HPLC reagent grade. Cyanuric chloride, ethanolamine, 2-picolyl-amine, and 8-hydroxyquinoline were purchased from Sigma-Aldrich (Sigma-Aldrich Chemie GmbH, 82024 Taufkirchen, Germany Melting points were determined with a Mel-Temp apparatus (Sigma-Aldrich Chemie GmbH, 82024 Taufkirchen, Germany)and are uncorrected. Magnetic resonance spectra (^1^H-NMR and ^13^C-NMR) were recorded on a JEOL 400 MHz spectrometer (JEOL, Ltd., Tokyo, Japan). Chemical shift values are reported in δ units (ppm). Elemental analyses were performed on a mod. 2400 elemental analyzer (PerkinElmer, Inc.940 Winter Street, Waltham, MA, USA), and the values found were within ±0.3% of the theoretical values. The compounds were named using ChemDraw Ultra version 14 (Cambridge Soft Corporation, Cambridge Park Dr, Cambridge, MA, USA). The chemical composition, the method of electrode preparation, the reference and the counter electrode are the same as used previously in our studies [39].

### 3.2. General Method for the Synthesis of 1,3,5-Triazine Derivatives

A solution of cyanuric chloride (**2**, 1.84 g, 10.0 mmol) in dioxane (50 mL) was slowly added to a solution of the nucleophile [8-hydroxyquinoline (**3**, 4.78 g, 33.0 mmol), ethanolamine (**5**, 2.01 g, 33.0 mmol), or 2-(aminomethyl)pyridine (**7**, 3.57 g, 33.0 mmol)] in dioxane (50 mL) at 0 °C. Anhydrous potassium carbonate (9.7 g, 70.0 mmol) was added to the reaction mixture at the same temperature. After complete addition of the K_2_CO_3_ the reaction mixture was stirred for 24 h at 80 °C. After cooling to room temperature, the reaction mixture was filtered and washed with hot dioxane. The solvent was removed under reduced pressure, and the residue was extracted with dichloromethane (2 × 50 mL, in case of preparation of **8** followed washing with H_2_O (20 mL). The organic extracts were combined and dried with anhydrous MgSO_4_. The pure product was obtained by recrystallization from dichloromethane–hexane (1:2) to afford the product **8** in pure state. In case of **4**, the product was obtained after evaporation of dioxane and the residue washed with water and the solid product recrystallized from ethyl acetate. In case of **6**, the product was obtained after evaporation of dioxane at room temperature to get a white solid product.

*2,4,6-Tris(Quinolin-8-yloxy)-1,3,5-triazine* (T3Q, **4**). White solid mp 268–271 °C; yield 86%; ^1^H-NMR (DMSO-*d*_6_, Figure S1): δ 7.46–7.55 (m, 9H, Ar; b,e,f); 7.81 (dd, *J* = 2.4, 2.0 Hz, 3H, Ar,d), 8.34 (dd, *J* = 1.6 Hz, 3H, Ar,*c*), 8.81(dd, *J* = 1.6, 1.6 Hz, 3H, Ar, a) ppm; ^13^C-NMR (DMSO-*d*_6_, Figure S1): δ 120.9, 122.1, 126.3, 126.2, 128.9, 136.0, 139.9, 147.0, 150.5, 173.6 ppm; Anal. Calc. for (C_30_H_18_N_6_O_3_; 510.51): C, 70.58; H, 3.55; N, 16.46; found C, 70.79; H, 3.64; N, 16.29.

*2,2′,2″-[(1,3,5-Triazine-2,4,6-triyl)tris(azanediyl)]tris(ethan-1-ol)* (T3EA, **6**). White solid mp 99 °C; yield 83% [40] mp 97–99 °C, 81.5% yield]; FT-IR: 3415 (OH), 3340 (NH), 1585–1478 (C=N) cm^−1^; ^1^H-NMR (DMSO-*d*_6_, Figure S2): δ 3.25 (brs, 6H, –NCH_2_–), 3.45 (brs, 6H, –CH_2_O), 4.57 (brs, 3H, 3OH), 6.35-6.55 (brt, 3H, NH) ppm; ^13^C-NMR (DMSO-*d*_6_, Figure S2): δ 42.7 (–NCH_2_), 60.3 (–CH_2_O–), 165.7 (C=N) ppm. MS: *m*/*z* = 259.47 [M]^+^ (Figure S3).

*N^2^,N^4^,N^6^-Tris(Pyridin-2-ylmethyl)-1,3,5-triazine-2,4,6-triamine* (T3AMPy, **8**). Off white solid mp 76–8 °C; yield 85% [41,42], yield 83%]; ^1^H-NMR (CDCl_3_): δ 4.46 (br, d, *J* = 41 Hz, 6H), 7.27–7.09 (br, m, 9H), 7.63 (br, d, *J* = 50 Hz, 3H), 8.43 (br, d, *J* = 17 Hz, 3H) ppm; ^13^C-NMR (CDCl_3_): δ 45.9 (–CH_2_–), 121.8, 121.9, 136.5, 148.9, 160.0, 166.1 (C=N) ppm.

### 3.3. Electrochemical Measurements

Polarization curves and EIS data were conducted using a Solartron 1470E (multichannel system, (Solartron, Armstrong Mall, Farnborough, Hampshire, UK)) with the Solartron 1455A as FRA. The polarization curves were recorded with a sweep rate of 1 mV/s. EIS measurements were measured in the frequency range of 0.01–10 kHz. Pt sheet was used as counter electrode while a calomel electrode was used as arefernce electrode. The working electrode was cut from steel rod with the following composition (wt: 0.14% C, 0.57% Mn, 0.21% P, 0.15% S, 0.37% Si, 0.06% V, 0.03% Ni, 0.03% Cr and Fe balance. The test solution used in all experiments was 1M HCl containing different concentrations of the investigated inhibitors (50–250 ppm).

## 4. Conclusions

The electrochemical results revealed that T3Q and TA3MPy have excellent corrosion protection performance towards the corrosion of steel in acidic chloride solution. The protection of steel occurs via adsorption of T3Q, T3AMPy and T3EA and blocking of the active sites on the steel surface. The electrochemical results revealed that the inhibition occurs by suppressing the anodic and cathodic reactions. The calculated values of *IE* from the *EIS* method follow the same trend as those obtained from the polarization results. The results obtained indicated that the more nitrogen atoms in the terminal groups of the inhibitor, the better the corrosion protection performance, so T3Q and T3AMP**y** gave better protection for steel than T3EA, which has an oxygen atom in its terminal side chain of the triazine moiety. Finally, the triazine moiety is not the only factor responsible for the inhibitory action, but also the structure of the side chain attached has a major effect for the corrosion inhibition.

## Figures and Tables

**Table 1:** The effect of T3Q, T3EA and T3AMPY concentrations on the IE% values for steel in acidic chloride solutions using electrochemical methods.

Inhibitor	Polarization Method						EIS Method		
	Conc. (ppm)	*Ba (mV)*	*Bc (mV)*	*E_corr_ (V)*	*i_corr_ μA/cm^2^*	*IE%*	*R_p_ Ohm*	*Cdl (μF/cm^2^)*	*IE%*
	Blank	69	120	−0.3955	839	____	1.80	334	____
**T3Q (4)**	50	55	131	−0.3337	55	93.4	28.5	109	93.6
	150	50	187	−0.3271	53	93.6	30	108	94.0
	250	77	103	−0.3463	14	98.8	100	90	98.2
**T3EA (6)**	50	98	631	−0.3315	557	33.6	2.8	198	35.6
	150	79	413	−0.3357	299	64.3	5.2	153	65.3
	250	63	174	−0.3352	125	85.1	13	125	86.1
**T3AMPy (8)**	50	56	170	−0.3324	63	92.4	24	112	92.5
	150	53	105	−0.3382	36	95.7	46	105	96.0
	250	50	112	−0.3348	34	96.9	47.5	104	96.2

## Supplementary Materials

Supplementary materials can be accessed at: http://www.mdpi.com/1420-3049/21/4/436/s1.
