# Supplementary material for: sym-Trisubstituted 1,3,5-Triazine Derivatives as Promising Organic Corrosion Inhibitors for Steel in Acidic Solution
Source: Molecules. 2016 Mar 31;21(4):436. doi: 10.3390/molecules21040436 (PMC6274514; doi:10.3390/molecules21040436)
Supplement: Supplementary file 1 [file molecules-21-00436-s001.pdf]

sym-Trisubstituted 1,3,5-Triazine Derivatives as Promising Organic Corrosion Inhibitors for Steel in Acidic Solution

Ayman El-Faham, Kholood A. Dahlous, Zeid A. AL Othman, Hamad A. Al-Lohedan and Gamal A. El-Mahdy

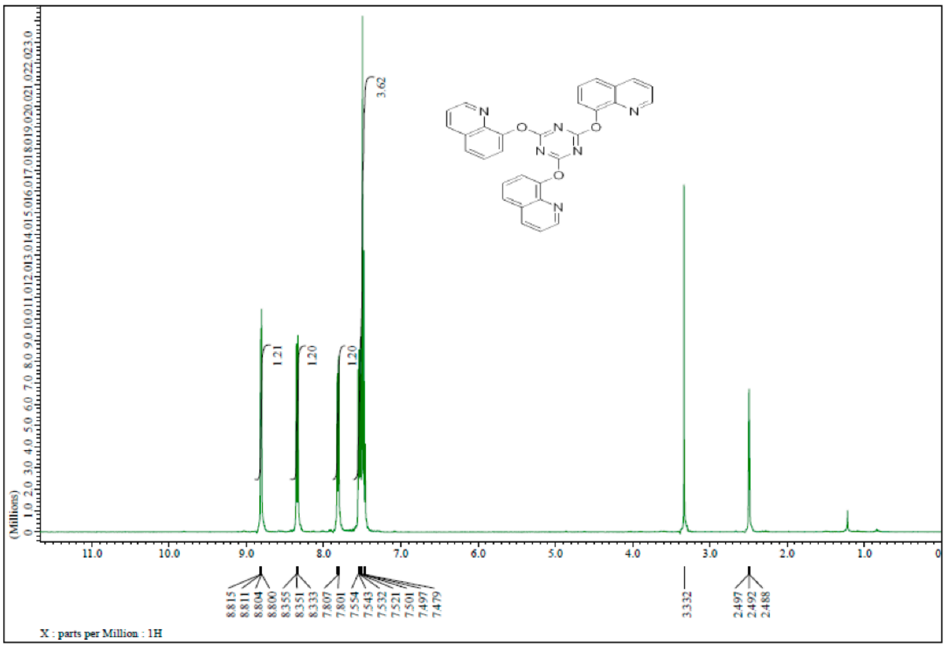

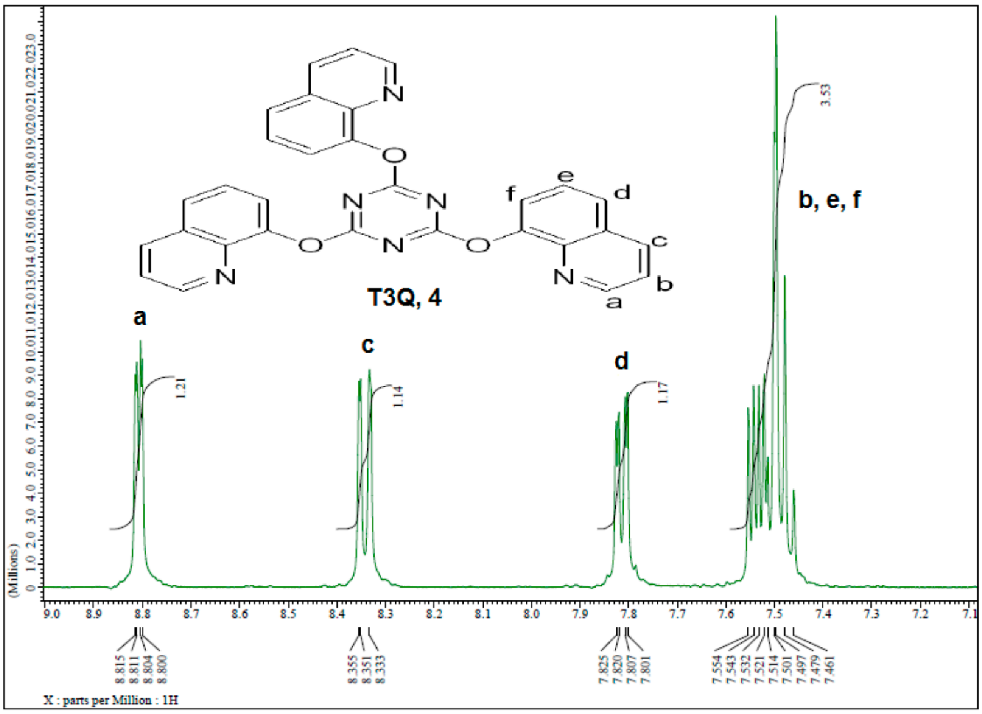

Figure S1. *Cont.*

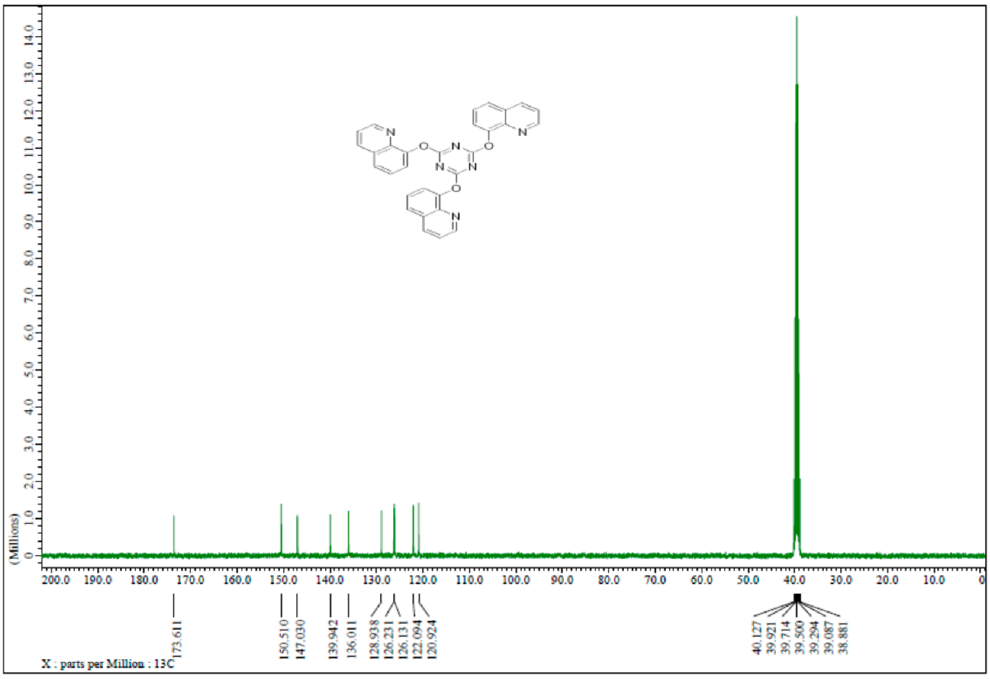

Figure S1. Expansion of ^{1}H-NMR and ^{13}C-NMR of T3Q (**4**).

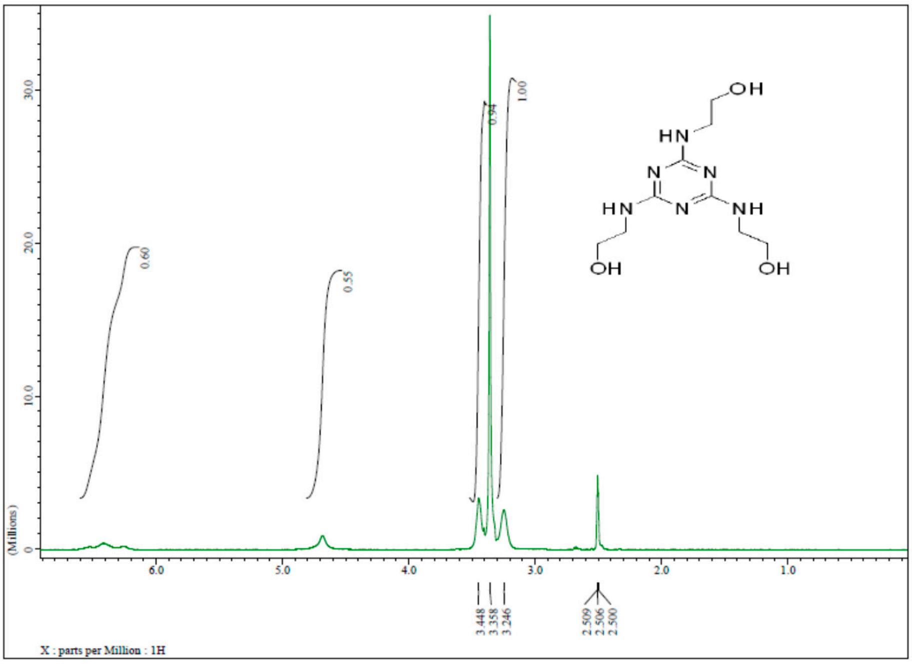

Figure S2. *Cont.*

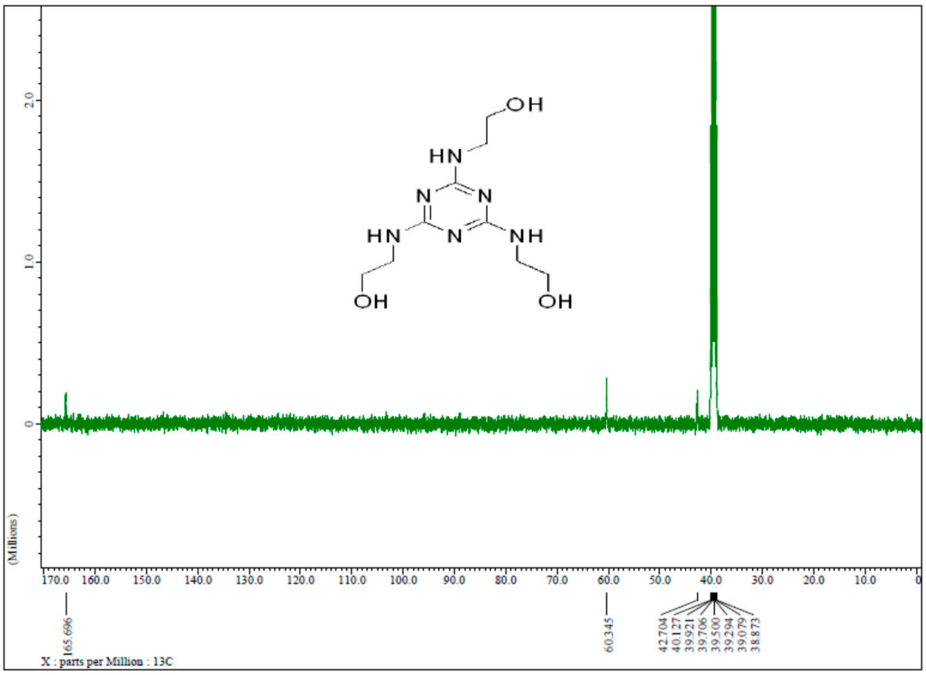

Figure S2. ^{1}H-NMR and ^{13}C-NMR of compound T3EA (**6**).

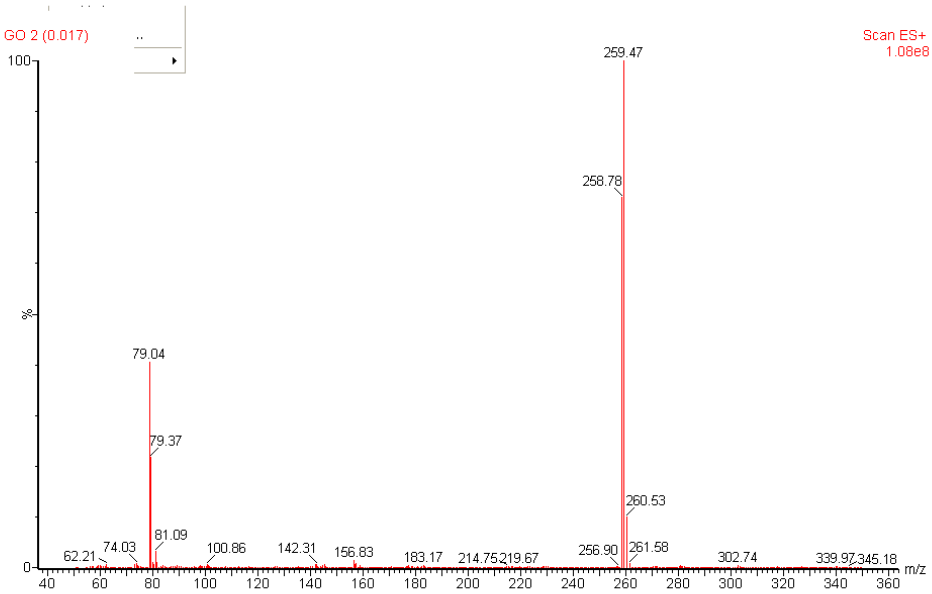

Figure S3. MS of compound T3EA (**6**).
